# MSC-NTF (NurOwn®) exosomes: a novel therapeutic modality in the mouse LPS-induced ARDS model

**DOI:** 10.1186/s13287-021-02143-w

**Published:** 2021-01-19

**Authors:** Haggai Kaspi, Jonathan Semo, Nathalie Abramov, Chen Dekel, Stacy Lindborg, Ralph Kern, Chaim Lebovits, Revital Aricha

**Affiliations:** Brainstorm Cell Therapeutics, Ltd., 1325 Avenue of Americas, New York City, NY 10019 USA

**Keywords:** Exosomes, Acute respiratory distress syndrome, COVID-19, Mesenchymal stem cells, Lung injury

## Abstract

**Background:**

One of the most severe complications of the current COVID-19 pandemic is acute respiratory distress syndrome (ARDS). ARDS is caused by increased amounts of pro-inflammatory cytokines, leading to lung damage and loss of lung function. There are currently no effective therapies for combatting ARDS. Mesenchymal stem cells (MSCs) have been suggested as a potential treatment for ARDS due to their significant immunomodulatory properties. MSC small extracellular vesicles (sEVs), including exosomes, modulate the immune response as effectively as MSCs themselves, with the added advantages of increased safety and tissue penetration.

**Methods:**

We isolated sEVs from MSCs induced to secrete increased levels of neurotrophic and immunomodulatory factors, termed Exo MSC-NTF, and compared their ability to treat ARDS, in a lung injury LPS mouse model, to sEVs isolated from naïve MSCs (Exo MSC). Measurments of lung histopathological changes and neutrophil infiltration, blood oxygen saturation, and bronchoalveolar lavge fluid (BALF) proinflammatory cytokines and coagulation related factors were performed.

**Results:**

We found that Exo MSC-NTF was superior to Exo MSC in reducing LPS-induced ARDS markers, including physiological lung damage such as alveolar wall thickness, fibrin presence, and neutrophil accumulation, as well as increasing oxygenation levels. Furthermore, Exo MSC-NTF reversed the imbalance in the host immune response, seen as decreased IFN-γ, IL-6, TNF-α, and RANTES levels in the bronchoalveolar lavage fluid.

**Conclusions:**

These positive preclinical results suggest that Exo MSC-NTF may be suitable as a therapy for COVID-19-induced ARDS and are more effective at combatting ARDS physiological, pathological, and biochemical symptoms than sEVs isolated from non-induced MSCs.

## Background

Acute respiratory distress syndrome (ARDS) is the most common and severe complication of the current COVID-19 pandemic [1]. In ARDS, an accumulation of inflammatory cells in the lungs releases large amounts of pro-inflammatory cytokines, known as a cytokine storm, causing widespread inflammation, pulmonary damage, loss of lung function, and death [2, 3]. Currently, there are no effective pharmacological treatments addressing the underlying mechanisms that cause ARDS [2] and all available treatments are supportive measures.

Bone marrow-derived mesenchymal stem cells (MSCs) are increasingly being evaluated for the treatment of ARDS and sepsis due to their immunomodulatory and regenerative properties [4]. MSCs are also capable of inhibiting the secretion of pro-inflammatory cytokines, such as TNF-α, IL-6, and IFN-γ, thereby potentially mitigating the ensuing cytokine storm [5]. Indeed, preliminary preclinical and clinical results have shown that MSCs can alleviate lung dysfunction in animal lung injury models [6], ARDS, and COVID-19 patients [7, 8]. The therapeutic effects of MSCs are exerted in part in a paracrine manner by releasing exosomes rather than local engraftment. Exosomes are nano-sized (30–120 nm) extracellular vesicles (EVs), secreted by different cell types, including MSCs. Exosomes participate in cell-to-cell communication by delivering various cargo, including miRNA, mRNA, lipids, and proteins from their cells of origin [9]. Compared to cellular treatment, MSC-derived exosomes are inherently safer for intratracheal administration and have several advantages, such as low immunogenicity, high stability, no potential to transdifferentiate into a different cell type, and enhanced tissue penetration capabilities [9]. Thus, MSC-derived exosomes are emerging as a cell-free alternative to cell-based therapy for ARDS [10]. A small clinical trial of COVID-19 patients treated with MSC exosomes has shown that one treatment increased patient oxygenation, reduced the immune response, and increased anti-inflammatory cytokine levels [11].

The precise mechanism by which MSCs and MSC exosomes exert their therapeutic effects in ARDS is not fully understood, but it is thought to involve a combination of anti-inflammatory and regenerative properties. The induction of MSCs to express high amounts of secreted growth factors increases their capability to treat tissue damage [12]. In this preclinical study, we used a proprietary process developed by Brainstorm, based on MSCs isolated from the total bone marrow sample, expanded and induced to differentiate into neurotrophic and immunomodulatory factors secreting MSCs (MSC-NTF), termed NurOwn [13]. The goal of this study was to investigate the treatment effect of small EVs (sEVs) derived from NurOwn MSC-NTF cells (Exo MSC-NTF) and sEVs derived from undifferentiated MSCs (Exo MSC) in the ability to treat pulmonary damage and inflammation in lipopolysaccharide (LPS)-induced ARDS in BALB/C mice. We surmised that Exo MSC-NTF might have increased abilities to decrease both clinical and tissue manifestation of ARDS due to their elevated levels of growth factors. Indeed, Exo MSC-NTF were superior to Exo MSC, in a murine model for LPS-induced lung inflammation, at increasing oxygen saturation, preventing lung tissue damage, and reducing lung inflammatory cytokine amount.

## Methods

### Cells

Bone marrow MSCs were isolated from a healthy volunteer (Lonza, Walkersville, MD, USA). After expansion, cells were cultured in a PBS mini bioreactor (PBS biotech, USA), loaded with 25 g of Synthemax II low concentration microcarriers (Corning, USA) and 10–15 million cells. Cells were cultured in high-glucose DMEM (Biological Industries, Israel) with 10% platelet lysate (PL), glutamine, sodium pyruvate, and heparin for 7 days. MSCs were characterized by phenotypic analyses of cell surface antigens by flow cytometry, as recommended by the International Society for Cellular Therapy [14]. For Exo MSC production, cells were cultured without PL for an additional 4 days, and the medium was harvested every 2 days. For Exo MSC-NTF, the PL-containing medium was switched to a differentiation medium, as described previously [15]. Briefly, MSCs were induced to differentiate into MSC-NTF cells (neurotrophic factors secreting MSCs) using a medium-based approach in which cells were incubated in a medium containing 1 mM dibutyryl cyclic AMP (cAMP), 20 ng/ml human basic fibroblast growth factor (hbFGF), 5 ng/ml human platelet-derived growth factor (PDGF-AA), and 50 ng/ml human Heregulin β1.

### Small EV isolation

Isolation of EVs from conditioned media was performed using tangential flow filtration (TFF) using the KrosFlo KR2i system (Repligen, USA) with 300 kDa MWCO PES hollowfiber (Repligen). In brief, conditioned media were concentrated 5-fold; the retentate was diafiltrated with 5 volumes of PlasmaLyte 148 (Baxter, UK) followed by an additional concentration of retentate. Finally, the retentate was sterile filtered.

### Experimental design

A total of 35 BALB/C female mice with LPS-induced ARDS were randomly assigned to receive Exo MSC, Exo MSC-NTF, or PlasmaLyte treatment through the intratracheal route of administration (IT, 800 μg of LPS—ChemCruz, 055:B5). Naive mice (*n* = 10, without LPS instillation) were injected with an equal volume of PBS. Treated animals received daily dose of 50 μl Exo MSC or Exo MSC-NTF via an endotracheal tube (2.0 × 10^10^ vesicles/ml). Treatment began 3 h after LPS administration for a total of 3 daily treatments. All animals were sacrificed 72 h after the LPS instillation. Control mice received 50 μl of PlasmaLyte at the same time points. sEVs and vehicle tubes were coded prior to administration and thus were not revealed to animal handlers.

Animals were measured daily for oxygen saturation and heart rate during the treatment period and hematology, lung histopathology, and bronchoalveolar lavage (BAL) fluid, serum, and differential cell counts by fluorescence-activated cell sorting (FACS).

### Animal procedures

Female, 8 weeks old, BALB/C mice were obtained from Envigo (Israel) and maintained in “Science in Action” (Ness Ziona, Israel) facility. Animal handling was performed according to guidelines of the National Institute of Health (NIH) and the Association for Assessment and Accreditation of Laboratory Animal Care (AAALAC). The experiment was performed under the approval by “The Israel Board for Animal Experiments” (approval number IL-20-6-225). Animals were weighed daily and were excluded from the study if body weight decreased by 20% from baseline or by more than 10% between measurement. In addition, animals were excluded from the study if any of the following was observed: severe dehydration, lack of movement, skin lesions, continuous tremor, or respiratory failure. Animals had free access to food and drinking water throughout the experiment.

IT administration of EVs was performed under isoflurane sedation. In parallel, blood oxygen levels were measured using MouseSTAT Jr. Pulse Oximeter for Mice & Rats (Kent Scientific). Briefly, mice were anesthetized using isoflurane and kept under anesthesia during monitoring. The hind paw of the mouse was placed in the paw sensor, with the pad directly over the red light. SpO2 levels were recorded for each mouse.

BALF was collected by intratracheal injection of 0.5 ml PBS with 0.1 mM EDTA followed by gentle aspiration for 3 times. Recovered fluid was pooled and centrifuged. The BALF supernatant was preserved for the measurement of cytokines and coagulation factors. The sediment cells were resuspended and subjected to FACS analysis.

To examine whether LPS IT administration was successful, we performed FACS analysis on BALF to observe changes in different leucocyte populations (T and B lymphocytes, eosinophils, neutrophils, dendritic cells, and monocytes/macrophages; data not shown). Since eosinophilia is one of the hallmarks of LPS inflammation, we excluded animals in which eosinophil percentage following LPS administration was < 35% (average ± SEM of eosinophils with or without LPS administration was 88.3 ± 0.9% and 18.7 ± 2.1%, respectively). Two animals from the LPS + plasmaLyte group and a single animal from the Exo MSC-treated group did not meet this criterion and were excluded from the study.

### EV characterization

Quantification and size distribution measurements of EVs were performed using the ZetaView nanoparticle tracking analyzer (Particle Metrix, Germany).

Characterization of EV membranal markers was performed with the MACSPlex exosomes kit (Miltenyi) with 7.5 × 10^8^ EVs per sample. The signal was read using CytoFlex FACS (Beckman Coulter).

### Transmission electron microscopy (TEM)

Exosomes were fixed in 20% paraformaldehyde/glutaraldehyde, loaded onto 200 mesh lacey Formvar carbon-coated grid that was blotted and plunged into liquid ethane using a Gatan CP3 automated plunger, and stored in liquid nitrogen until use. Frozen specimens were transferred to Gatan 914 cryo-holder and maintained at temperatures below − 176 °C inside the microscope. Samples were inspected with a Tecnai G2 microscope (FEI—Teramo fisher) with an acceleration voltage of 120 kV, which is equipped with a cryobox decontaminator. Images were taken using digital micrograph (Gatan) in different resolutions.

### Histology

Lungs were harvested and fixed in 4% formaldehyde. The tissues were then trimmed in a standard position and put in embedding cassettes. One cassette was prepared per animal. Paraffin blocks were sectioned at ~ 4 μm thickness, put on glass slides, and stained with hematoxylin and eosin (H&E). Pictures were taken using an Olympus microscope (BX60, serial NO. 7D04032) at objective magnification of × 4 and × 10 and microscope’s Camera (Olympus DP73, serial NO. OH05504).

A quantitative analysis for acute lung injury (ALI) was performed using a severity scoring scale of 0–2, based on the American Thoracic Society Documents, 2011 [16]. Analysis was performed by a certified veterinarian pathologist (Patho-logica Ltd., Ness Ziona, Israel) who was blinded to experimental treatment.

*Neutrophils*: Not visible within the field—a score of 0; 1–5 neutrophils—1; more than 5 neutrophils—2.

*Fibrin*: Not visible within the field—a score of 0; a single well-formed band of fibrin within the airspace—1; multiple eosinophilic membranes—2.

*Thickened alveolar walls:* Due to technical artifacts, only septal thickening that is equal or greater than twice normal was considered. Less than × 2—score 0; × 2–× 4—score 1; more than × 4—score 2.

The analysis was based on measurements of 20 fields, using objective magnification of × 4 and × 10 (HPF).

Neutrophil cell count was performed using MATLAB color-based, brightness-based, and morphological-based segmentation. The cells were counted from a rectangle of 88,892 μm^2^.

### Cytokine multiplex measurements

BALF cytokine concentrations were measured using ProcartaPlex Luminex platform (ThermoFischer, USA). The measurements were performed in duplicates (25 μl each) with a custom multiplex panel detecting the following mouse cytokines: IFNγ, TNFα, RANTES, IL-6, IL-10, IL-1α, IL-1β, IP-10, MIP1α, and MCP-1. Measurements were performed using Luminex MAGPIX instrument, and results were analyzed with Xponent 4.2 software according to manufacturer instructions.

### ELISA

BALF thrombin–antithrombin and tissue factor were measured using ELISA kits (abcam, UK—ab137994 and ab214091 respectively) according to manufacturer protocol.

### Analysis of EV protein cargo

To measure the content of specific proteins in sEVs, 1 ml of sEV enriched fractions was precipitated using ExoQuick-CG (SBI, USA). EV pellets were lysed using M-PER Mammalian Protein Extraction Reagent (ThermoFischer, USA), supplemented with 1:200 Protease Inhibitor Cocktail Set III, EDTA-Free (Calbiochem). Following 10-min incubation in room temp, the lysates were frozen and thawed twice to ensure complete lysis. Lysates’ protein concentrations were measured using BCA kit (ThermoFischer, USA) and concentrations of 60–75 μg/ml were used for ELISA assays. Amphiregulin (AREG) and LIF concentrations were measured using Quantikine kits (R&D Systems, Minneapolis, MN; Cat# DAR001, DLF00B). HGF and TSG-6 concentration were measured with ELISA kits from RayBiotech, USA (Cat# ELH-HGF-CL-1, ELH-TSG6-1). Signals were quantified using Sunrise plate reader and the Magellan Software V7.2 (Tecan, Switzerland).

### In vitro immunomodulation assay

The immunomodulatory properties of Exo MSC and Exo MSC-NTF were evaluated in vitro by examining inhibition of cytokine secretion by peripheral blood mononuclear cells (PBMCs) in response to activation with phytohemagglutinin (PHA). PBMCs (5 × 10^5^) were stimulated with 10 μg/ml PHA and incubated with Exo MSC or Exo MSC-NTF (2 × 10^9^ particles) for 4 days in culture. IFNγ and TNFα were measured in the culture supernatant using a commercial ELISA (DuoSet ELISA, R&D Systems, Minneapolis, MN) that was read at 450 nm with Sunrise plate reader and analyzed by the Magellan Software V7.2 (Tecan, Switzerland).

### Statistical analyses

Statistical analyses were performed using GraphPad Prism 7 software (GraphPad Software, San Diego, CA). For analysis of cytokine concentrations, TAT and tissue factor ELISAs, and neutrophil count, one-way ANOVA followed by Tukey’s post hoc were performed. Histological scorings were analyzed using Kruskal–Wallis followed by Dunn’s post hoc.

Oxygen saturation was analyzed using repeated measurements two-way ANOVA followed by Tukey’s post hoc.

## Results

**Fig. 1:**
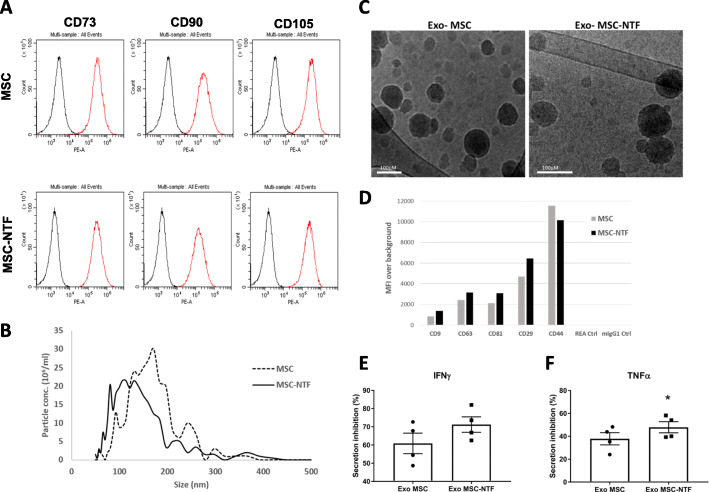
Analysis of MSC and MSC-NTF cells and their derived EVs. **a** FACS analysis of the MSC markers CD73, CD90, and CD105 on MSC and MSC-NTF cells. **b** Nanoparticle tracking analysis of naïve Exo MSC and Exo MSC-NTF. Exo MSC had a median size of 146 nm, and Exo MSC-NTF had a median size of 114 nm. **c** Transmission electron microscopy image of Exo MSC and Exo MSC-NTF. Scale bar represents 100 nm. **d** MACSPlex exosome kit FACS analysis of exosome expression of tetraspanins (CD9, CD63, CD81), MSC (CD44, CD29), and isotype controls (REA Ctrl, mIgG1 Ctrl) in Exo MSC and Exo MSC-NTF. **e**, **f** Immunomodulatory activity of the sEVs as determined by inhibition of IFNγ and TNFα secretion by activated PBMCs. Cell culture supernatant ELISA was performed following incubation with EVs from four independent donors relative to untreated activated PBMCs. Mean ± SEM, *p < 0.05 paired *t* test

MSCs were induced to differentiate into MSC-NTF cells using a culture medium-based process. MSC-NTF cells maintained the original MSC immunophenotype, whereby > 95% of the population expressed CD73, CD90, and CD105 (flow cytometry analysis, Fig. 1a). Small extracellular vesicles (sEVs) were isolated from the culture medium of MSC and MSC-NTF cells derived from the same donor. Nanoparticle tracking analysis (NTA) revealed that naïve MSC sEVs (Exo MSC) had a median size of 146 nm and MSC-NTF sEVs (Exo MSC-NTF) had a median size of 114 nm (Fig. 1b). Similar particle sizes were also observed using transmission electron microscopy (Fig. 1c). In general, the average median size of Exo MSC-NTF was not different from Exo MSC, when comparing sEVs isolated from several different donors (data not shown).

To confirm that the isolated sEV samples are exosome enriched, we performed FACS analysis, using the MACSPlex exosome kit, to check for exosomal surface marker expression. We found all three hallmark tetraspanins markers (CD9, CD63, CD81) were expressed at similar levels in both Exo MSC and Exo MSC-NTF samples (Fig. 1d). In addition, both samples expressed the MSC markers CD44 and CD29 but not several hematopoietic markers (e.g., CD45, CD4), consistent with reported expression analyses [17].

To evaluate the immunomodulatory capacity of the sEVs, Exo MSC or Exo MSC-NTF were added to activated PBMCs. This resulted in inhibition of IFNγ and TNFα secretion (Fig. 1e, f). While there was no significant difference in the ability of Exo MSC and Exo MSC-NTF to inhibit IFNγ secretion, Exo MSC-NTF were significantly more efficient in inhibiting TNFα secretion.

**Fig. 2:**
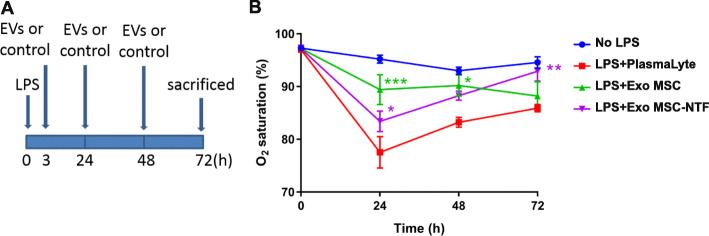
Exo MSC-NTF increase O_2_ saturation in an LPS lung injury mouse model. **a** Experimental setup. Mice received intratracheal (IT) treatment of 800 μg LPS followed by three treatments with Exo MSC, Exo MSC-NTF, or vehicle (PlasmaLyte), 3 h, 24 h, and 48 h after LPS exposure. **b** O_2_ saturation 24 h, 48 h, and 72 h following LPS/vehicle injection. Mean ± SEM, *n* = 9–13. *p < 0.05, **p < 0.01, ***p < 0.001 vs. LPS + PlasmaLyte group. Repeated measurements two-way ANOVA followed by Tukey’s post hoc

Administration of lipopolysaccharide (LPS) to mice induces severe lung damage and is a prevalent ARDS animal model [18] (see study design in Fig. 2a). To assess the physiological effects of Exo MSC and Exo MSC-NTF in the ARDS model, we measured blood oxygen saturation daily. Oxygen saturation was reduced in LPS-treated groups and was significantly improved by both Exo MSC and Exo MSC-NTF (Fig. 2b).

**Fig. 3:**
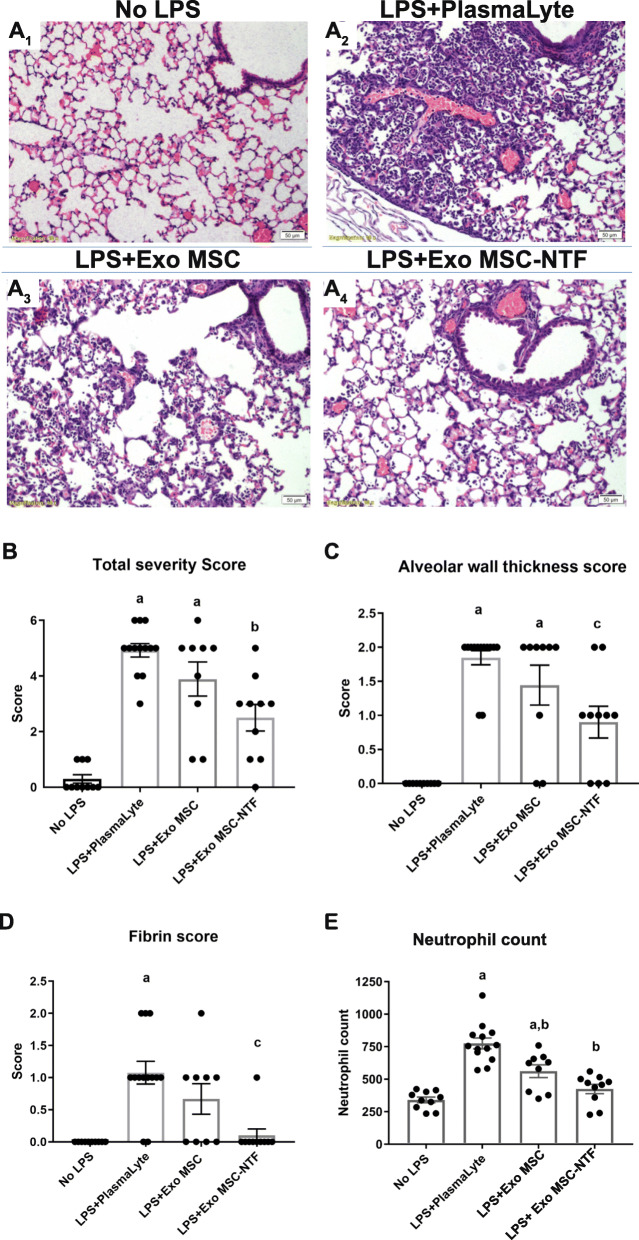
Exo MSC-NTF mitigates pathological lung effects due to LPS administration. **a** Lung histological sections of (1) healthy controls (no LPS), (2) LPS exposed treated with vehicle (PlasmaLyte), and LPS exposed treated with (3) Exo MSC or (4) Exo MSC-NTF. Quantification of lung damage according to American Thoracic Society documents: **b** severity score, **c** alveolar wall thickness, and **d** fibrin accumulation. **e** Neutrophil count in lung tissue. Mean ± SEM, *n* = 9–13. Twenty fields per animal were examined (**b**-**d**). ^a^*p* < 0.05 vs. no LPS control; ^b^*p* < 0.05 vs. LPS + PlasmaLyte; ^c^*p* ≤ 0.01 vs. LPS + PlasmaLyte. Kruskal–Wallis followed by Dunn’s post hoc (4**b**-**d**) and one-way ANOVA followed by Tukey’s post hoc (**e**)

Histological analysis of lung sections showed significant lung damage 72 h after LPS treatment (Fig. 3a). Exo MSC-NTF significantly alleviated the LPS-induced physical damage, as did Exo MSC, albeit to a lesser extent (compare Fig. 3a_3_ to a_4_). Lung damage was quantified according to the criteria set forth by the American Thoracic Society [16], assessing alveolar wall thickness, fibrin presence, and neutrophil accumulation which sums together to a total severity score. Treatment with Exo MSC-NTF significantly lowered the total severity score as compared to untreated LPS animals (Fig. 3b; mean score of 2.5 vs. 4.5), but Exo MSC treatment did not (mean score of 3.9). Exo MSC-NTF, but not Exo MSC, significantly reduced both wall thickness (Fig. 3c) and fibrin accumulation (Fig. 3d) following LPS treatment. We further analyzed lung sections for neutrophil accumulation and found that treatment with Exo MSC-NTF reduced the LPS-induced neutrophil accumulation to a level comparable to a healthy control (Fig. 3e). Exo MSC also reduced neutrophil count, but less efficiently.

**Fig. 4:**
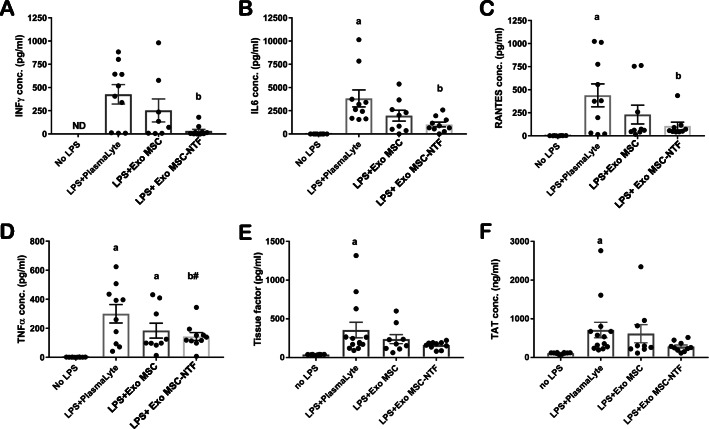
Exo MSC-NTF repress several LPS-induced immune effects in the bronchoalveolar lavage fluid (BALF) of LPS-treated mice. Quantification of the immune response in the BALF of treated mice. Measurements of **a** IFN-γ, **b** IL-6, **c** RANTES, and **d** TNF-α using ProcartaPlex platform. A measure of coagulation by **e** tissue factor and **f** thrombin–antithrombin complex (TAT) using ELISA. Mean ± SEM, *n* = 9–10 (ProcartaPlex) or 9–13 (ELISA). ^a^*p* < 0.05 vs. no LPS control, ^b^*p* < 0.05 vs. LPS + PlasmaLyte, ^b#^*p* = 0.058 vs. LPS + PlasmaLyte. One-way ANOVA followed by Tukey’s post hoc

To understand the factors that contributed to reduced lung damage and increased blood oxygen saturation following EV treatment, we measured biochemical changes in bronchoalveolar lavage fluid (BALF). MSC exosomes have extensive immunosuppressive and immunomodulatory capabilities [19] and have been proposed as a treatment for ARDS and COVID-19 [20], as potential modulators of the severe cytokine storm. We examined the expression of ten cytokines (IFNγ, IL-6, IL-10, RANTES, TNFα, IL-1β, IL-1α, MCP-1, IP-10, and MIP-1α) and found that Exo MSC did not significantly reduce BALF expression of any of them. However, Exo MSC-NTF reduced IFNγ (Fig. 4a), IL-6 (Fig. 4b), and RANTES (Fig. 4c) BALF levels. Levels of BALF TNFα showed a tendency towards a decrease (*p* = 0.058, Fig. 4d), while the other cytokines were not significantly affected by Exo MSC-NTF (data not shown).

Increased coagulation is a prominent feature of ARDS [21] and is correlated with COVID-19 disease severity [22]. To determine if EV treatment affected coagulation, we measured the levels of tissue factor (TF), a mediator of coagulation, and thrombin–antithrombin complex (TAT), a measure of coagulation, in BALF. While the effects did not reach statistical significance compare to PlasmaLyte control, we found a tendency of Exo MSC-NTF to reduce both TF (2.24-fold, Fig. 4e) and TAT levels (2.5-fold, Fig. 4f), while the effect of Exo MSC was milder (1.5-fold and 1.15-fold decrease, respectively, Fig. 4e, f). Interestingly, there was no statistical difference between Exo MSC and MSC-NTF-treated mice and healthy controls.

**Fig. 5:**
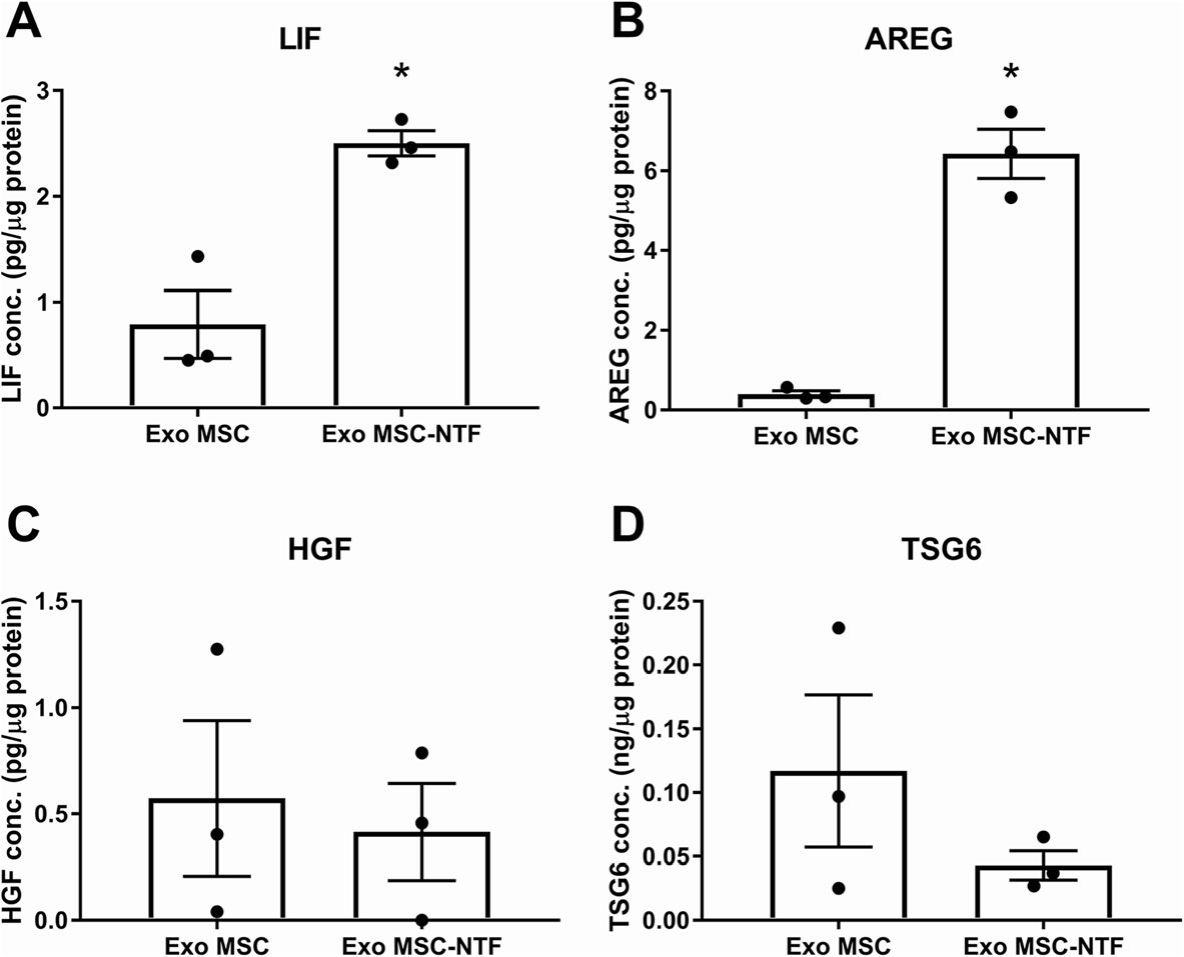
Differences in protein cargo between Exo MSC-NTF and Exo MSC. ELISA of Exo MSC and Exo MSC-NTF lysates from three independent donors displayed higher abundance of **a** LIF and **b** AREG in Exo MSC-NTF. **c** HGF and **d** TSG-6 were detected in both Exo MSC and Exo MSC-NTF but without significant differences. Mean ± SEM, *n* = 3, *p < 0.05 paired *t* test

To explore differences between Exo MSC and Exo MSC-NTF which might contribute to the superior effect of Exo-NTF treatment, we evaluated differences in protein cargo of Exo MSC and Exo MSC-NTF from three independent donors. We focused on proteins which (i) we had previously identified to be upregulated in MSC-NTF cells in comparison to naïve MSCs (data not shown), (ii) were previously reported in EV database ExoCarta [23] to be loaded into EVs, and (iii) were reported to have a beneficial effect in lung injury or ARDS models. The abundance of four proteins was thereafter measured in EV lysates. ELISA measurements revealed that AREG was 16-fold more abundant and LIF was > 3-fold more abundant in Exo MSC-NTF in comparison to Exo MSC (Fig. 5a, b; *p* = 0.013 and *p* = 0.015, respectively). In addition, HGF and TSG-6 were found to be present in both types of EVs, but without significant differences (Fig. 5c, d).

In summary, we demonstrated that Exo MSC-NTF is a promising and innovative biological therapy for ARDS. IT administered Exo MSC-NTF significantly improved lung histology and function, increased blood oxygen saturation, and reduced inflammatory cytokines and coagulopathy biomarkers. Exo MSC also demonstrated an improvement yet reduced over Exo MSC-NTF.

## Discussion

The predominant pattern of lung pathology in patients with COVID-19 patients is diffuse alveolar damage, similar to that described in patients afflicted with ARDS. COVID-19-induced ARDS is a type of respiratory failure associated with widespread inflammation and dysregulated cytokine production demonstrated in both serum and BALF. Compared to patients with moderate COVID-19, patients with severe/critical infections have much higher levels of inflammatory cytokines, particularly interleukin IL-6, IL-1β, and TNF-α, in their BALF and lung tissue [24]. Increased neutrophil counts have also been associated with COVID-19 disease severity and poor prognosis, and autopsies show extensive neutrophil infiltration of pulmonary capillaries. The presence of platelet–fibrin thrombi in small arterial vessels is consistent with coagulopathy, which appears to be common among COVID-19 patients [25]. Neutrophil extracellular traps (NETs) have been shown to exert thrombogenic activity through the expression of functionally active tissue factor (TF) [26, 27].

LPS lung instillation is one of the most used rodent models for ARDS. This model shares many pathological features with COVID-19-related ARDS, such as hypoxemia, neutrophil accumulation, alveolar space thickening, fibrin and TF pathology, and high levels of inflammatory cytokines [18]. The similarities between the LPS-treated rodents and COVID-19 patients, in terms of lung damage and the inflammatory response, make LPS a reliable model to evaluate potential COVID-19 therapies. In this study, we demonstrated significant improvement in the relevant ARDS parameters following treatment with Exo MSC-NTF.

Damage to the endothelial membrane and pulmonary vasculature allows the accumulation of coagulation factors within the alveoli. TF exposed on the surface of damaged endothelial cells, macrophages, and monocytes promote fibrin formation. High levels of inflammatory factors activate neutrophils to form NETs and amplify macrophage and monocyte surface TF exposure [28]. MSCs reduce acute lung injury in the LPS-ARDS model through NET inhibition [29], suggesting a promising therapeutic approach in COVID-ARDS [30]. In this study, we demonstrated that Exo MSC-NTF reduced neutrophil count, TF, and fibrin, in the lung tissue, thereby interrupting a disease cascade that may explain the early lung recovery or the prevention of damage following intratracheal exosome treatment.

The differences in therapeutic efficacy between Exo MSC and Exo MSC-NTF raises the possibility they carry different cargo proteins which are responsible for the differential effect. In this study, we measured the expression of 4 proteins, of which LIF and AREG were found to be significantly increased in Exo MSC-NTF compared to Exo MSC. It was previously demonstrated that LIF takes part in attenuating lung damage and inflammation in multiple models, including LPS [31], viral infection [32], and *E. coli* infection [33]. For example, intratracheal co-injection of LIF with LPS was shown to reduce neutrophil infiltration and BAL pro-inflammatory cytokine levels [31]. Recently, the possible beneficial effect of LIF administration to COVID-19 patients was also discussed [34].

AREG is a factor in the epidermal growth factor family and was previously shown to promote repair in LPS-induced ALI: administration of AREG neutralizing antibodies worsens lung injury [35], whereas AREG administration ameliorated lung injury [36].

Therefore, the improved outcomes of mice treated with Exo MSC-NTF may be, at least in part, the result of increased lung delivery of factors such as LIF and AREG. However, additional factors may play a role in the superior beneficial effect by Exo MSC-NTF.

## Conclusions

The positive results of intratracheal Exo MSC-NTF in improving lung function and lung pathology and in re-balancing the immune response in the ARDS model suggest that this therapeutic modality may have the potential for coronavirus pneumonia as well as for other causes of ARDS.

## Data Availability

The datasets used and/or analyzed during the current study are available from the corresponding author on reasonable request.

## Abbreviations

AREG: Amphiregulin
ARDS: Acute respiratory distress syndrome
BALF: Bronchoalveolar lavage fluid
DMEM: Dulbecco’s modified Eagle medium
EV: Extracellular vesicle
FACS: Fluorescence-activated cell sorting
IT: Intratracheal
LPS: Lipopolysaccharide
MiRNA: MicroRNA
MCP1: Monocyte Chemoattractant Protein-1 / CCL2
MSC: Mesenchymal stem cells
NETs: Neutrophil extracellular traps
NTA: Nanoparticle tracking analysis
PBS: Phosphate-buffered saline
PL: Platelet lysate
RANTES: CCL5, a cytokine
TAT: Thrombin–antithrombin complex
TF: Tissue factor
TSG-6: TNF-Stimulated Gene 6 Protein / TNFAIP6
